# Case report: epidermoid inclusion cyst of the clitoris as a long-term complication of female genital mutilation

**DOI:** 10.1186/s13256-019-2035-6

**Published:** 2019-04-27

**Authors:** Ozer Birge, Mustafa Melih Erkan, Aliye Nigar Serin

**Affiliations:** 1 Department of Gynaecology and Obstetrics, Nyala Sudan Turkey Training and Research Hospital, Nyala, Darfur Sudan; 2 Department of Gynaecology and Obstetrics, Kent Hospital Bayrakli Medical Center, Izmir, Turkey

**Keywords:** Female genital mutilation type III, Infibulation, Epidermoid cyst, Clitoris, Complication

## Abstract

**Background:**

Female genital mutilation is a common procedure in sub-Saharan Africa that causes serious short- and long-term complications. Although physicians can overcome these complications sometimes, they can be very confusing to diagnose. In this report, we discuss the surgical management of a patient with an epidermal inclusion cyst of the clitoris as a long-term complication of type III female genital mutilation.

**Case presentation:**

A healthy 43-year-old African woman who was a nonsmoker and nonalcoholic presented with a large genital mass causing difficulty in urination and sexual discomfort. The patient had three full-term spontaneous vaginal deliveries without any complications. Perineal examination revealed a 6 × 10-cm, well-circumscribed, mobile, nontender, rounded cystic swelling in the right periclitoral area that was obstructing the urinary meatus and vaginal introitus. A surgical procedure was performed for total excision of the clitoral mass. Pathological findings showed an epidermoid inclusion cyst.

**Conclusions:**

Besides increasing clinicians’ awareness of female genital mutilation and its long-term complications, public education campaigns should be designed to eradicate this practice.

## Background

The World Health Organization (WHO) describes female genital mutilation (FGM) as “all procedures that involve partial or total removal of the external female genitalia, or other injury to the female genital organs for non-medical reasons” [1]. Despite the lack of evidence for any health benefits of this procedure and its recognition as a violation of human rights by the WHO, approximately 100 million women are estimated to have undergone FGM worldwide, and FGM is practiced in more than 30 countries of West Africa, the Middle East, and Southeast Asia [2]. The perpetuation of FGM is most commonly due to cultural and religious beliefs; however, it is not restricted to any ethnic, religious, or socioeconomic class. The widespread and continuous immigration of these women into Western societies presents a diagnostic challenge to health care professionals in developed countries and necessitates preparedness for the management of the acute and long-term complications of FGM.

FGM is classified into four types by the WHO. Type I is excision of the prepuce with or without excision of part or all of the clitoris. Type II is excision of the prepuce and clitoris with partial or total excision of the labia minora. Type III is excision of part or all of the external genitalia and stitching/narrowing of the vaginal opening (infibulation). Type IV includes all other procedures with the aim of tightening or narrowing, such as pricking, piercing, or incision of the clitoris or labia; cauterization by burning of the clitoris and surrounding tissues; scraping of the vaginal orifice; cutting of the vagina; introduction of corrosive substances or herbs into the vagina to cause bleeding. Types I and II comprise most of the cases (up to 80%), whereas type III represents the most severe and extreme form and sometimes is referred to as “pharaonic circumcision” [3]. All types of FGM are associated with multiple, sometimes life-threatening, acute and long-term complications, including hemorrhage, shock, infection, dysmenorrhea, infertility, recurrent urinary tract infections, obstetric sequelae, clitoral scarring and cysts, and psychological and sexual problems [4].

In this report, we describe a case of a multiparous female adult with a histologically confirmed large epidermal inclusion cyst of the clitoris who had a history of type III FGM performed at the age of 8 years.

## Case presentation

An otherwise healthy 43-year-old African Sudanese-Darfurian woman presented with a large genital mass causing difficulty in urination and sexual discomfort. The patient first noticed this “sometimes itchy mass” when it was very “tiny” while she was a teenager, and then it started growing over the last 10 years. The patient, who was married at the age of 16, had three full-term spontaneous vaginal deliveries at home without any complications. In her past medical history, there were no other chronic diseases or surgeries, except for FGM. She is living in a village with a large extended family and not working professionally. She does not consume alcohol and or smoke. The patient decided to seek medical attention at her husband’s request because she has not been able to tolerate coitus over the last 5 years because of the pain caused by the mass, which has been negatively affecting their family life because they wish to conceive again. The patient has a history of type III FGM when she was 8 years old. The FGM was performed by a nonmedical traditional practitioner in her village, without any additional history of trauma or surgery. After genital mutilation, she did not have any problem, and for this mass she had received no other treatment for years.

**Fig. 1:**
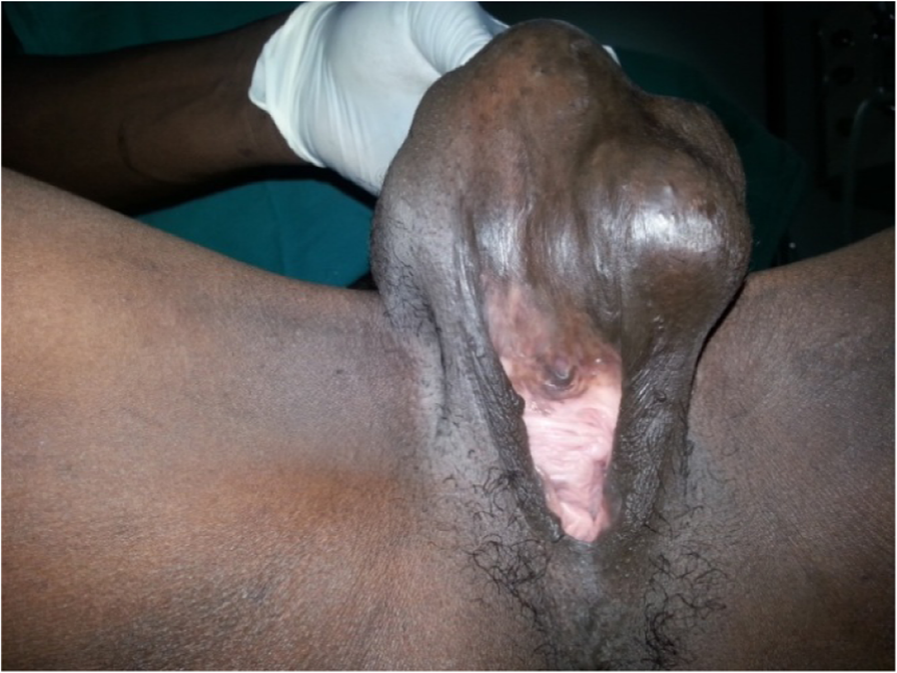
Preoperative inspection of vulvar region

**Fig. 2:**
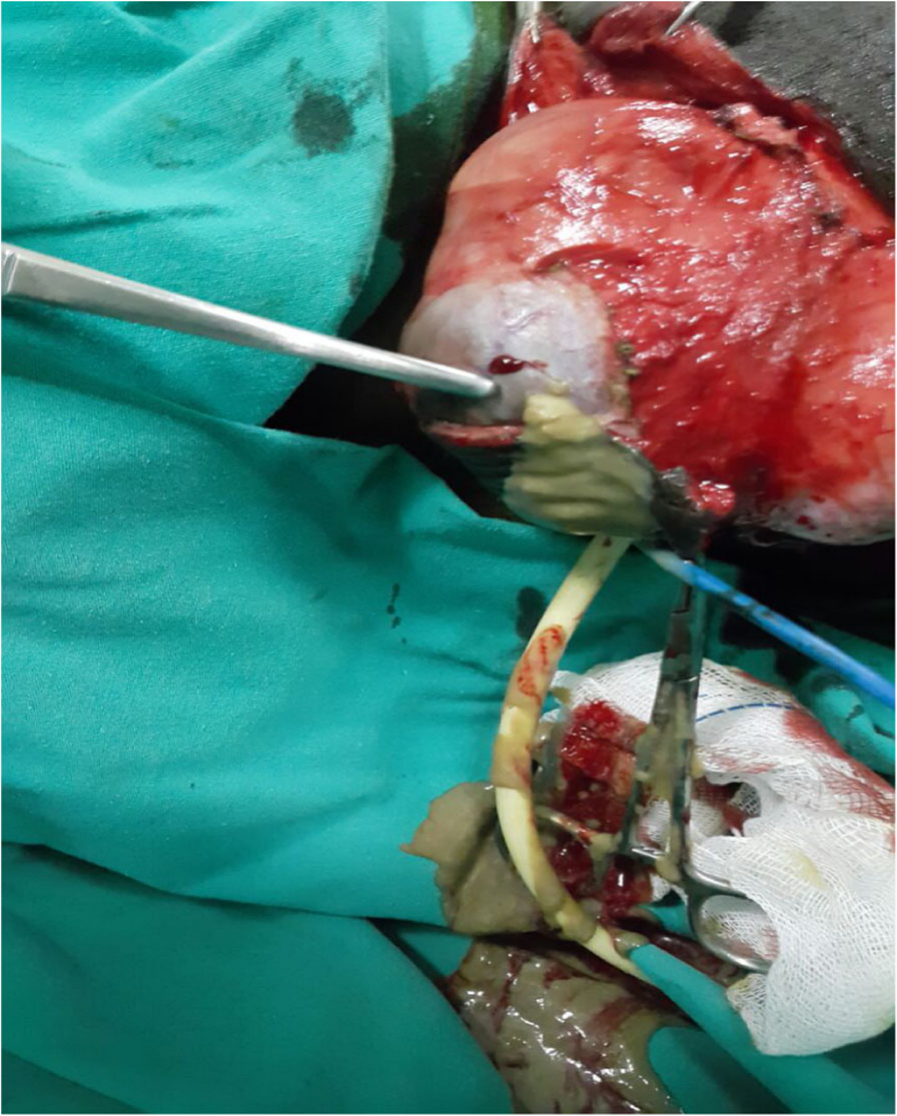
Intraoperative appearance of the cyst (include off-white grumous, keratinous material)

**Fig. 3:**
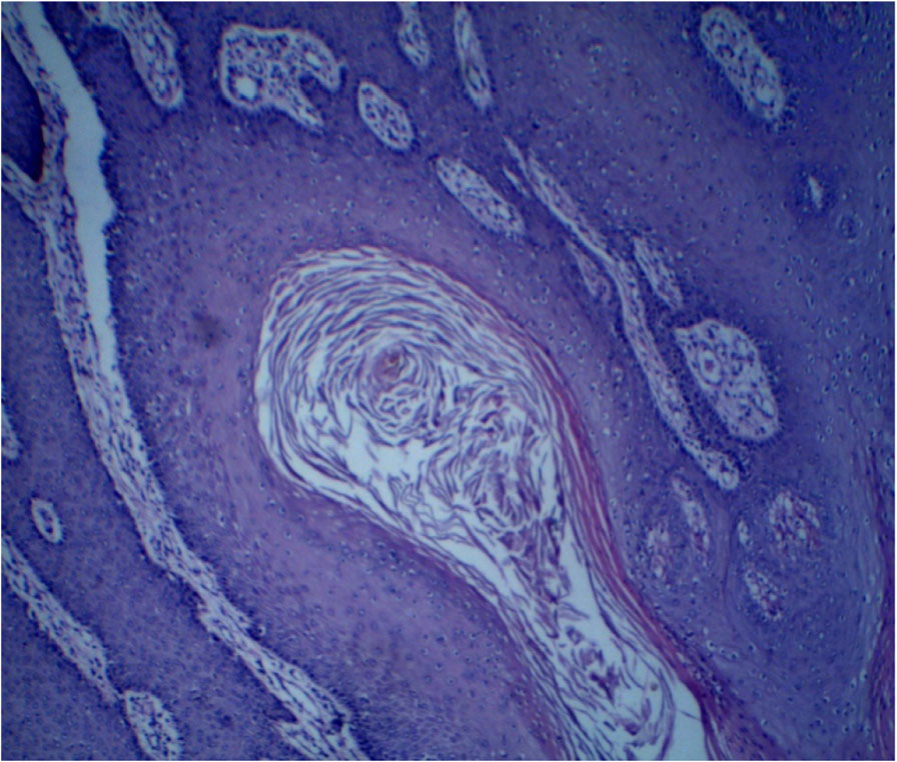
Histopathological appearance of the epidermoid cyst lined by stratified squamous epithelium and filled with keratinous material (H&E stain, magnification × 40)

When she was admitted to our hospital, her blood pressure was 110/72 mmHg, and her body temperature was 36.8 °C. Her cardiac rhythm was regular, and all pulses were palpated normal. Her physical examination revealed no pathological findings other than the genital mass. The result of her neurological examination was normal. Perineal examination revealed a 6 × 10-cm, well-circumscribed, mobile, nontender, rounded, cystic swelling in the right periclitoral area that was obstructing the urinary meatus and vaginal introitus. The multilobulated mass was along the line of the previously performed type III FGM scar (Fig. 1). The rest of the examination was normal. Ultrasound imaging suggested benign lobulations and septations in a cystic swelling. After informed consent was obtained, an elective surgery was performed with the patient under general anesthesia for total excision of the clitoral mass. Intraoperative findings included the presence of a well-demarcated, encapsulated subcutaneous cystic mass with a volume of 6 × 7 × 10 cm, filled with dark yellow “cheesy” keratinous material (Fig. 2). In the microscopic examination, the cyst had a squamous epithelial wall with evidence of keratinous material in the lumen, and therefore it was diagnosed as an epidermoid inclusion cyst (Fig. 3). The postoperative period was without any complications, and by the third-month visit, the patient had no anatomic or functional problems in the perineal region. The patient stated that the complete resolution of her complaints improved her quality of life as well as her relationship with her husband. In her follow-up, by the sixth and ninth visits, the operative side was clear, and there was not any hypertrophic scar tissue. The patient told us that she had not had any pain during intercourse after her surgery.

## Discussion

This case is very rare and unique. In the literature, there are many case reports about epidermoid inclusion cysts of the clitoris, but these are usually secondary to aesthetic procedures and traumas or congenital cysts that can mimic clitoromegaly. There is only one reported series of 32 patients whose cases were secondary to FGM. In that series, the mean diameter of cysts upon examination was 4.2 ± 2 cm, and the mean duration of hospital admission after FGM was 5 ± 4 years. In our patient, the mass could be classified as a huge mass obstructing the introitus with a 10-cm diameter.

Nearly all major medical professional organizations consider the practice of FGM as a violation of human rights, owing to its severe and potentially life-threatening acute and long-term physical and psychological complications. However, FGM is currently practiced in about 30 countries in Africa and in some countries in Asia and the Middle East. In addition, albeit secretly, certain immigrant communities in North America and Europe continue to perform this procedure, where it is mainly done by traditional practitioners and nonmedical personnel [5–8]. Approximately 100 million to 150 million women worldwide are estimated to have undergone FGM, with a daily incidence of 6000 African girls aged 4 to 12 years old, and 2 million girls per year are thought to be at risk of undergoing such a procedure [5, 7, 9]. Although types I and II FGM comprise 80% of the FGM practices worldwide, the more extreme type III FGM is the most commonly performed procedure in Sudanese women, with an overall FGM prevalence of 89% in the region [10]. This report describes a case of an epidermal clitoral inclusion cyst as a long-term complication of type III FGM (also known as *infibulation*) in an adult female from the Darfur region of Sudan, Africa.

Epidermal inclusion cysts result from proliferation of epidermal cells within a circumscribed space, forming a cystic mass after the implantation of keratinized epidermal elements in the dermis. These benign lesions are usually due to vulvar skin trauma followed by an epidermal invagination and proliferation. They grow slowly and usually do not cause symptoms, but they may become infected, causing pain and discomfort [11]. They rarely reach a size to obstruct the entire urethral meatus and vaginal orifice. Multiparous Sudanese women usually have a heavily deformed perineum with intensive scarring due to repetitive episiotomies, deinfibulations, and reinfibulations, and therefore they more frequently present with inclusion cyst formations [12]. In addition to the physical complications, inclusion cysts may cause psychological problems, including disfigurement, shame, and fear of cancer, and they may affect the personal and family life of the patient. Our patient was a 43-year-old multiparous Sudanese woman with complete obstruction of the urethral and vaginal orifices due to a large epidermoid cyst causing sexual discomfort approximately 35 years after a type III FGM procedure (infibulation). The surgical excision of the mass resolved her symptoms, as well as improved her quality of life and her relationship with her husband.

## Conclusions

It is well established that FGM can cause devastating short- and long-term complications. The recognition of this serious problem is very important for health care professionals, considering the increased exposure to FGM worldwide resulting from emigration and globalization. It is our hope that increasing understanding and awareness of FGM will help physicians to better serve patients similar to our patient described in this report.
